# Primary Cutaneous Rosai-Dorfman Disease of the Scalp

**DOI:** 10.5826/dpc.1101a86

**Published:** 2020-12-07

**Authors:** Haruki Mizuta, Eiji Nakano, Naoya Yamazaki

**Affiliations:** 1 Department of Dermatologic Oncology, National Cancer Center Hospital, Tokyo, Japan; 2 Department of Plastic and Reconstructive Surgery, Graduate School of Medicine, Osaka City University, Osaka, Japan; 3 Division of Dermatology, Department of Internal Medicine, Kobe University Graduate School of Medicine, Hyogo, Japan

**Keywords:** Rosai-Dorfman disease, cutaneous Rosai-Dorfman disease, scalp, angiosarcoma

## Introduction

Rosai-Dorfman disease (RDD) is a reactive and histioproliferative disorder of unknown cause. It predominantly affects the cervical lymph nodes, with extranodal lesions in 40% of cases (skin lesions comprise 16%). The presence of characteristic RDD symptoms (fever, night sweats, and weight loss), termed B symptoms, is considered systemic RDD. Primary cutaneous RDD (CRDD) comprises 3% of all RDDs. It is localized to the skin with no systemic symptoms or abnormal blood examination findings. Although it often occurs on the face, there are very few reports of primary CRDD of the scalp. We report a case of primary CRDD of the scalp that needed to be distinguished from angiosarcoma.

## Case Presentation

**Figure 1:**
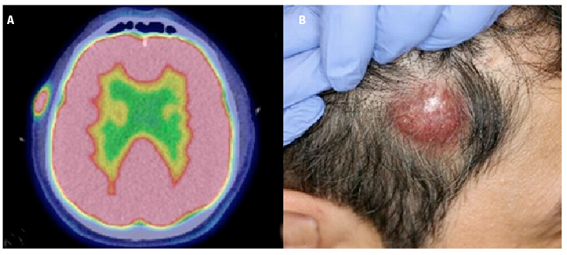
Clinical findings: (A) PET/CT taken at the nearby hospital. A skin lesion with an accumulation is seen in the right temporal region away from the skull. (B) Clinical findings at the first visit: a smooth, soft, and mobile red mass measuring 2.5 × 2 cm with no surrounding purpura.

A 51-year-old man noticed a mass on his head 6 months before his first visit, and 2 months prior, biopsy had been performed at a nearby hospital owing to gradual mass enlargement. Histopathological findings indicated angiosarcoma. PET/CT detected a skin lesion with accumulation in the right temporal region and no metastasis (Figure 1A). Therefore, he was referred to our hospital. Clinical examination revealed a smooth, soft, and mobile red mass measuring 2.5 × 2 cm in the right temporal region (Figure 1B), and the pathological diagnosis was reconsidered because he was younger than the typical angiosarcoma patient and had no purpura around the mass.

**Figure 2:**
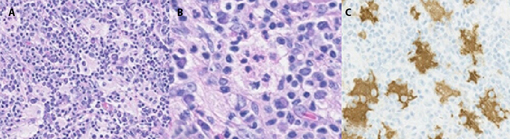
Histopathological features: (A) There are many large histiocytes forming a nodular/diffuse pattern (H&E, ×200). (B) Normal lymphocytes and neutrophils are detected in the cytoplasm of the large histiocytes, which is called emperipolesis (H&E, ×400). (C) The large histiocytes are positive for S100 (immunohistochemical staining, ×200).

Numerous inflammatory cells (plasma cells) were seen in the dermis (Figure 2A). Many large histiocytes formed a nodular/diffuse pattern (Figure 2B).

Normal lymphocytes and neutrophils were detected in the cytoplasm of large histiocytes, termed emperipolesis. Tumor cells were positive for S100 and CD68 and negative for CD1a, CD34, and ERG. (Figure 2C). Thus, CRDD was diagnosed. At follow-up 10 months after the patient’s first visit, with no treatment, the tumor reduced in size.

## Conclusions

Five cases of CRDD of the scalp have been reported (4 primary skull RDD with subcutaneous infiltration and 1 primary CRDD of the scalp) [1]. Accumulation of RDD on PET/CT has been reported [2]. Therefore, it must be differentiated from other malignant diseases.

Our case mimicked an atypical angiosarcoma that presented as classical angiosarcoma found on sun-damaged skin of the head and neck of elderly patients, lymphedema-associated angiosarcoma in postmastectomy or lymphatic malformations, and post-radiation angiosarcoma in radiation fields.

Histopathological CRDD findings revealed large histiocytes, which form nodular/diffuse patterns with skin infiltration and are positive for S100 and CD68 but negative for CD1a.

These histological features indicate RDD as a non-Langerhans cell histiocytosis. Further, immunohistochemical staining of CD34 and ERG, markers of vascular endothelium, distinguishes CRDD from angiosarcoma. Emperipolesis, in which normal lymphocytes and neutrophils are recognized in the cytoplasm of histiocytes, is a characteristic feature of CRDD. These 3 features must be present to suspect angiosarcoma. CRDD undergoes spontaneous regression and has a good prognosis. Minimally invasive modalities, such as excision and topical, local injection of, and internal use of steroids, are options for treatment; however, a benign or malignant diagnosis greatly influences the treatment strategy. Thus, it is crucial to consider CRDD in the diagnosis of atypical skin cancers.
